# Mortality assessment of patients with pancreatic cancer in Mexico, 2000–2014

**DOI:** 10.3332/ecancer.2017.788

**Published:** 2017-12-07

**Authors:** Omar González-Santiago, Myrna L Yeverino-Gutiérrez, María del Rosario González-González, Ruth Corral-Symes, Pilar C Morales-San-Claudio

**Affiliations:** Autonomous University of Nuevo León (UANL), School of Chemical Sciences, PhD in Pharmacy, San Nicolás de los Garza, Nuevo León 66455, Mexico

**Keywords:** cancer, mortality rate, pancreas, Mexico

## Abstract

The five-year survival rate remains low for pancreatic cancer (PC). The objective of this study was to describe PC mortality rates in Mexico and its seven socioeconomic regions. The data for the deaths were obtained from the National Institute of Statistics and Geography databases. The adjusted rates were calculated using the world standard population. During the study period, the average mortality rate was 4.29 per 100,000 inhabitants. By gender, the rate was 4.35 and 4.29 per 100,000 inhabitants for men and women, respectively. Socioeconomic region 7 had the highest mortality rates. There was a significant decline in mortality rates in all of the groups.

## Introduction

Pancreatic cancer (PC) continues to be one of the malignancies with the worst prognosis once it has been diagnosed. The survival of people who suffer from it is only 5% at five years. This is attributed mainly to the delay in its diagnosis, since in the initial phases, it presents with nonspecific symptoms that make it difficult to diagnose such as jaundice, weight loss, abdominal pain and osteomalacia. Only 10%–20% of patients are diagnosed in the early stages when tumour resection and a possible cure are feasible [1].

There are multiple risk factors for the development of PC, including both environmental and genetic factors. Smoking falls into the first category. It is one of the main risk factors and therefore a preventable cause of PC, as are diets high in meats and fats, low levels of folate, obesity, diabetes mellitus, chronic pancreatitis, alcoholism, infection from *Helicobacter pylori* and a family history [2, 3].

According to data from GLOBOCAN 2012, PC causes more than 331,000 deaths per year, which places it among the top ten causes of cancer death [4]. In developing countries, it may even be the fourth leading cause of death from cancer and in developed countries such as the United States, it is expected to be the second leading cause of death from cancer by 2030 [5].

In the case of Mexico, cancer is the third leading cause of death, with cardiovascular diseases and complications of diabetes mellitus occupying the top two places [6]. Given the importance of this type of cancer, the objective of this study was to describe the PC mortality trend in Mexico by gender, age and socioeconomic region.

## Methods

### Data

PC deaths were obtained from the databases of the National Institute of Statistics and Geography (INEGI), which are freely accessible through its web portal. International Classification of Diseases (ICD-10) code C25 was used, which corresponds to malignant tumour of the pancreas. Information about the population of Mexico for the years 2000, 2005 and 2010 was obtained from the population censuses also reported by INEGI. The population for the remaining years was obtained by direct interpolation of the respective population censuses. The data used can be accessed at https://figshare.com/articles/Raw_data_Mortality_assessment_of_patients_with_pancreatic_cancer_in_Mexico_2000_2014/5645788.

### Statistical analysis

The deaths were grouped by gender, three age groups (<40, 40–59 and >60 years) and socioeconomic region. There are seven socioeconomic regions in Mexico that range from the lowest (region 1) to the highest level of wealth (region 7), as classified by INEGI. The states that make up each of the regions are as follows: Region 1: Chiapas, Guerrero; Region 2: Campeche, Hidalgo, Puebla, San Luis Potosí, Tabasco, Veracruz; Region 3: Durango, Guanajuato, Michoacán, Tlaxcala, Zacatecas; Region 4: Colima, Mexico, Morelos, Nayarit, Querétaro, Quintana Roo, Sinaloa, Yucatan; Region 5: Baja California, Baja California Sur, Chihuahua, Sonora, Tamaulipas; Region 6: Aguascalientes, Coahuila, Jalisco, Nuevo León; and Region 7: Mexico City.

The adjusted mortality rate for each group was calculated per 100,000 inhabitants using the direct method and the world standard population. The difference between the means of the groups was analysed with the Student’s *t*-test. The mortality trend was estimated with a logarithmic regression of the adjusted rates and it was evaluated with the Student’s *t*-test where the alternative hypothesis is the slope not equal to 0. The annual percentage change (APC) was estimated with the following formula: APC = (e^m^ - 1) * 100 where *m* is the slope that was previously obtained in the logarithmic regression [7, 8]. NCSS version 11 statistical software was used for the analyses.

### Ethical considerations

The present study did not require the approval of the ethics committee because it is a retrospective study that used freely and publicly accessible data. INEGI databases do not allow or publish the identity of individuals.

## Results

During the study period, a total of 50,748 deaths due to PC were recorded. This represents 0.64% of all deaths in Mexico (Table 1), where the female gender, the 40–59 age group and the inhabitants of socioeconomic region 7 had the highest mortality percentages (0.77%, 0.79% and 0.77%, respectively).

On the other hand, the average adjusted mortality rate during the period was 4.29 per 100,000 inhabitants. By gender, the rate was 4.35 and 4.29 per 100,000 inhabitants for men and women, respectively (Table 2). The >60 age group and the inhabitants of region 7 had the highest mortality rates (27.1 and 6.1 per 100,000 inhabitants, respectively). The individuals that had lower rates were those <40 and the inhabitants of socioeconomic region 1 (0.12 and 3.04 per 100,000 inhabitants, respectively)

**Figure 1.**
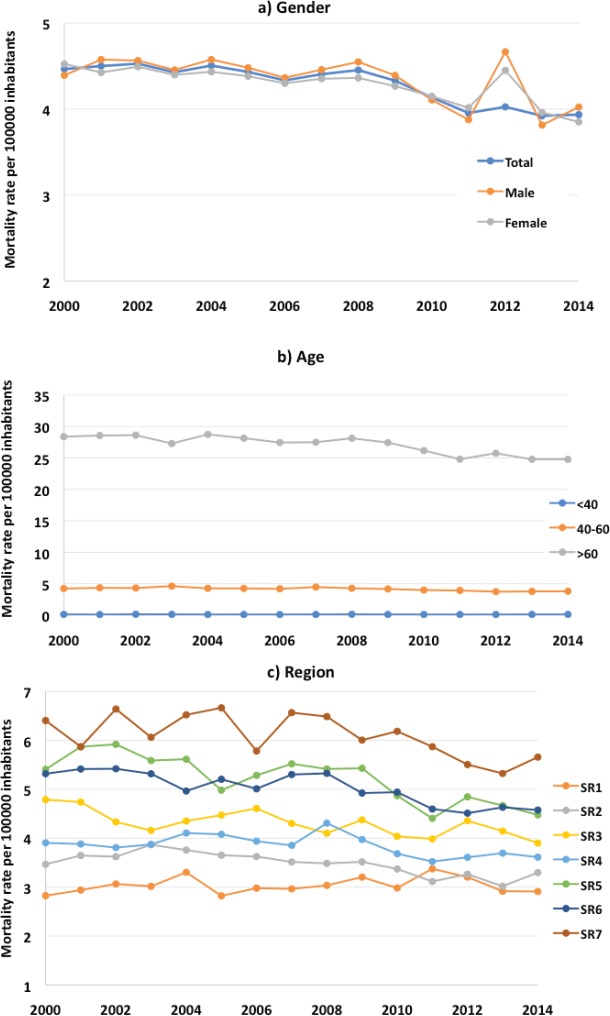
Pancreas mortality rates in Mexico: a) gender, b) age and c) socioeconomic region.

The temporal behaviour of the mortality rates by gender, age and socioeconomic region is presented in Figure 1. In all the groups studied, a downward trend in mortality rates is observed. With the exception of the <40 age group and the inhabitants of region 1 (p = 0.50 and p = 0.39, respectively), the rest of the groups had significant declines (Table 2). In general, the APC was −1.08%. By gender, this was −0.87 and −0.89 for men and women, respectively. The age group <40 had the lowest APC while the groups aged 40–60 and >60 had similar APC (−1.09% and −1.08%, respectively). By socioeconomic region, the inhabitants of region 5 had the highest APC (−1.74%), while those in region 1 had the lowest APC (0.3%).

## Discussion

In this study, PC mortality in Mexico was analysed. Previous studies in Mexico have shown that this type of cancer is in the sixth position in cancer mortality rates in men and the seventh in women [9]. Worldwide, the PC mortality rate is highly variable. Our results are similar to those of the population of China and Hong Kong, below those of the United States and some countries in Europe [10], and above those of Iran [11]. The causes of these differences at an international level are not well defined and more studies are needed in this area.

In terms of mortality by gender, several studies have reported a significant difference between men and women, with the former having higher mortality rates [12]. It should be noted that in our case there were no statistically significant differences (p = 0.48), and the causes of this result are not clear, as in Mexico the prevalence of smoking, one of the main risk factors, in men is almost double that of women [13]. It is likely that other associated factors that could contribute to this observation are the higher prevalence of obesity (75.6% vs 69.4%) and diabetes (10.3% vs 8.4%) in women [14].

The wealthiest socioeconomic regions in Mexico have the highest mortality rates. As previously mentioned, the wealthiest and therefore the most developed countries are those with the highest mortality rates. It is likely that this global pattern also applies at a regional level in each country. In addition, in developed countries, the proportion of elderly people is higher and if we consider that age is an important factor for the development of PC, this could explain in part the higher mortality rate in Mexico’s socioeconomic region 7. In this region, the proportion of elderly people (age >65) is higher than in the rest of the regions.

Unlike other countries such as Korea, Spain, France and Russia [10], in which there have been significant increases in mortality rates for this type of cancer, the results in this study showed a significant decline (p < 0.01) in mortality rates. Our results are consistent with what has been observed in countries like Iran, the United States and the United Kingdom [10, 11]. In the case of Mexico, this decrease could be due to improvements in PC detection techniques and greater access to treatments.

## Conclusion

The average PC mortality rate in Mexico for the period 2000–2014 was 4.29 per 100,000 inhabitants and showed a significant downward trend. Socioeconomic region 7 had the highest mortality rates.

## Conflicts of interest

The authors declare that there is no conflict of interest.

## Author contributions

Conception, design: OGS

Acquisition of data, statistical analysis: MLYG, MRGG

Interpretation of results: all authors

Manuscript writing and revising: RCS, PCMSC

Manuscript review: all authors

## Figures and Tables

**Table 1.** Total pancreatic cancer deaths in Mexico.

Variable		Pancreatic cancer	Total deaths	% of total
Total		50,748	7,936,677	0.64
Gender				
	Male	23,883	4,421,511	0.54
	Female	26,863	3,510,803	0.77
Age				
	< 39	1,287	1,575,865	0.08
	40–59	11,845	1,498,001	0.79
	> 60	37,575	4,824,822	0.78
Socioeconomic region				
	1	3,774	775,916	0.49
	2	8,408	1,579,108	0.53
	3	6,672	977,489	0.68
	4	9,743	1,553,078	0.63
	5	6,500	964,121	0.67
	6	8,262	1,134,256	0.73
	7	7,345	952,709	0.77

**Table 2.** APC in PC mortality rate.

Variable		Period		(Annual percentage change)	P value trend
		2000	2014		
Total		4.46	3.94	−1.08	<0.01
Gender					
	Male	4.39	4.02	−0.89	0.01
	Female	4.52	3.85	-0.87	<0.01
Age					
	<40	0.13	0.13	0.4	0.50
	40–60	4.24	3.81	−1.09	<0.01
	>60	28.39	24.78	−1.08	<0.01
Region					
	1	2.83	2.91	0.3	0.39
	2	3.47	3.30	−1.17	<0.01
	3	4.79	3.90	−1.02	<0.01
	4	3.91	3.61	−0.65	0.04
	5	5.41	4.48	−1.74	<0.01
	6	5.32	4.57	−1.25	<0.01
	7	6.40	5.66	−1	0.01
